# Immunotherapy of mesothelioma: the evolving change of a long-standing therapeutic dream

**DOI:** 10.3389/fimmu.2023.1333661

**Published:** 2024-01-08

**Authors:** Luana Calabrò, Giuseppe Bronte, Federica Grosso, Luigi Cerbone, Angelo Delmonte, Fabio Nicolini, Massimiliano Mazza, Anna Maria Di Giacomo, Alessia Covre, Maria Fortunata Lofiego, Lucio Crinò, Michele Maio

**Affiliations:** ^1^ Department of Translational Medicine, University of Ferrara, Ferrara, Italy; ^2^ Department of Oncology, University Hospital of Ferrara, Ferrara, Italy; ^3^ Department of Clinical and Molecular Sciences (DISCLIMO), Università Politecnica Delle Marche, Ancona, Italy; ^4^ Clinic of Laboratory and Precision Medicine, National Institute of Health and Sciences On Ageing (IRCCS INRCA), Ancona, Italy; ^5^ Mesothelioma, Melanoma and Sarcoma Unit, Azienda Ospedaliera SS Antonio e Biagio e Cesare Arrigo, Alessandria, Italy; ^6^ Department of Medical Oncology, IRCCS Istituto Romagnolo Per Lo Studio Dei Tumori (IRST) “Dino Amadori”, Meldola, Italy; ^7^ IRCCS Istituto Romagnolo Per Lo Studio Dei Tumori (IRST) “Dino Amadori”, Meldola, Italy; ^8^ Center for Immuno-Oncology, Medical Oncology and Immunotherapy, Department of Oncology, University Hospital of Siena, Siena, Italy; ^9^ Center for Immuno-Oncology, University of Siena, Siena, Italy; ^10^ EPigenetic Immune-Oncology Consortium Airc (EPICA), Siena, Italy; ^11^ Fondazione Network Italiano per la Bioterapia dei Tumori (NIBIT) Onlus, Siena, Italy

**Keywords:** pleural mesothelioma, immunotherapy, immune checkpoint inhibitors, adoptive cell therapy, epigenetic drugs

## Abstract

Pleural mesothelioma (PM) is an aggressive and rare disease, characterized by a very poor prognosis. For almost two decades, the world standard treatment regimen for unresectable PM has consisted of a platinum-based drug plus pemetrexed, leading to an overall survival of approximately 12 months. The dramatic therapeutic scenario of PM has recently changed with the entry into the clinic of immune checkpoint inhibition, which has proven to be an effective approach to improve the survival of PM patients. The aim of the present review is to provide a comprehensive overview of the most promising immunotherapeutic-based strategies currently under investigation for advanced PM.

## Introduction

Pleural mesothelioma (PM) is a rare, fatal disease that origins from the pleural membranes lining the lungs. According to the World Health Organization (WHO) 2015 histological classification, PM has been distinguished into three main morphological subtypes: epithelioid, characterized by a better prognosis, sarcomatoid, that has the most aggressive clinical behavior, and biphasic type that shows features of both epithelioid and sarcomatoid histology (1, 2). More recently, molecular and epigenetic findings have been also included in the most recent classification of PM (WHO 2021) (3). Although the use of asbestos was banned decades ago in most countries, the global rate of PM continues to increase slowly, due to previous, mostly occupational, asbestos exposure (4). A peak of PM is expected to be reached in the next 5 and 20 years in Western and Eastern countries respectively, due to the current use of asbestos in the latter. Despite these epidemiological landscape, treatment of PM did not significantly changed in the past 15 years, and the combination of cisplatin or carboplatin and pemetrexed has been set as the reference therapeutic scheme (5) for the majority of unresectable PM patients. However, the antitumor efficacy of this regimen remains unsatisfactory, as the median overall survival (mOS) of treated patients ranges between 12 and 14 months, and the 5-years survival is achieved by less than 5% of them. Due to its relevant role in the PM biology, angiogenesis has represented for many years a promising, largely investigated therapeutic target. Consistently, in 2016, the MAPS phase 3 clinical trial, has shown that the combination of Bevacizumab and Cisplatin-Pemetrexed regimen, significantly improves OS over standard chemotherapy (HR 0,77) with a mOS of 18,8 months (6). However, this regimen is presently utilized only in France for selected PM patients. In addition, plenty of trials have subsequently failed to demonstrate the therapeutic efficacy of targeting angiogenesis with both antibodies and multitargeted small molecule inhibitors in unselected PM subjects (7).

In this dark scenario, a flash of light, comes from immunotherapy. Indeed, in the last decade, the development of the immune-checkpoint inhibitors (ICI) has dramatically redesigned the therapeutic landscape of various tumor types, including PM. Consistently, dual ICI with the anti-cytotoxic T lymphocyte antigen (CTLA)-4 monoclonal antibody (mAb) ipilimumab plus the anti-programmed cell death protein (PD)-1 mAb nivolumab has proven greater efficacy than platinum-based regimen in first-line PM patients, becoming the new standard of care in several countries (8). Prompted by these results, an impressive development of ICI-based regimens is currently ongoing in any setting of PM.

In this review, we will critically discuss the most recent strategies with ICI-and non-ICI-based immunotherapeutic approaches currently under investigations for PM patients.

## Role of tumor microenvironment in the rationale for PM immunotherapy

Accumulating data have supported for years the notion that PM is an immunologically cold tumor, as it is characterized by a low median tumor mutational burden (TMB) (< 2 non-synonymous mutations *per* megabase), with less than 2% of patients harboring a TMB higher than 10 mut/megabase (9, 10), and a tumor microenvironment (TME) which is not overtly immune-infiltrated. Indeed, PM pathogenesis generally includes a prolonged inflammation caused by asbestos fibers, which ultimately affect the immune cell composition of the TME, with a high number of immunosuppressive cells among tumor infiltrating lymphocytes (TILs), and a low number of cytotoxic T-cells. The main immunosuppressive components of PM microenvironment include tumor-associated macrophages (TAMs) and myeloid-derived suppressor cells (MDSCs); conversely, tumor suppressive subpopulations are mostly represented by cytotoxic T lymphocytes, NK and B cells. Recently, evidences have demonstrated that components of the TME may influence the response to immunotherapy and model the genomic characterization through clonal selection (11, 12).

### Tumor-associated macrophages

TAMs are usually analyzed through flow cytometry of pleural effusions or immunohistochemistry of PM tissue. These cells reach an amount between a quarter and a half of all cells in the immune infiltrate (13, 14). TAMs are usually characterized by the expression of CD163, and in other cancer types they were demonstrated to reach the TME through the peripheral blood, thus deriving from circulating monocytes rather than from tissue-resident macrophages (15). Mesothelioma cells attract monocytes in the TME though releasing some chemokines, such as chemokine (C-C motif) ligand 2 (CCL2), chemokine (C-C motif) ligand 4 (CCL4), chemokine (C-C motif) ligand 5, and C-X-C motif chemokine ligand 12 (CXCL12). CCL2, which is the main chemoattractant, acts via CCR2 (16)). Chemokine receptors, such as CXCR4, CCR5, and CXCR1, were expressed in monocytes from pleural and peritoneal effusions of mesothelioma patients. Other receptors, such as CX3CR1 and CCR1, were upregulated in murine asbestos-induced mesothelioma (17, 18).

Monocytes and macrophages acquire a tumor suppressing phenotype through various molecules secreted by mesothelial cells, e.g., macrophage-colony stimulating factor (M-CSF) and interleukin (IL)-34, which have been detected in pleural effusions (16, 19). Furthermore, for the specific activation of macrophages, transforming growth factor beta (TGF-β) and IL-10 have also been identified, in addition to the pleural effusions, also in the supernatant from mesothelioma cultures and in mesothelioma tissue samples via immunohistochemistry (13, 20, 21).

The co-cultures with immunosuppressive macrophages favored mesothelioma cells to proliferate to a great extent and reduced their sensitivity to chemotherapy. An orthotopic syngeneic and immunocompetent mouse model of mesothelioma corroborated the relevant role of macrophages in the promotion of mesothelioma. The selective removal of the local macrophage population via clodronate encapsulated liposomes, reduced the number and invasiveness of the primary tumor and metastases (22).

**Figure 1:**
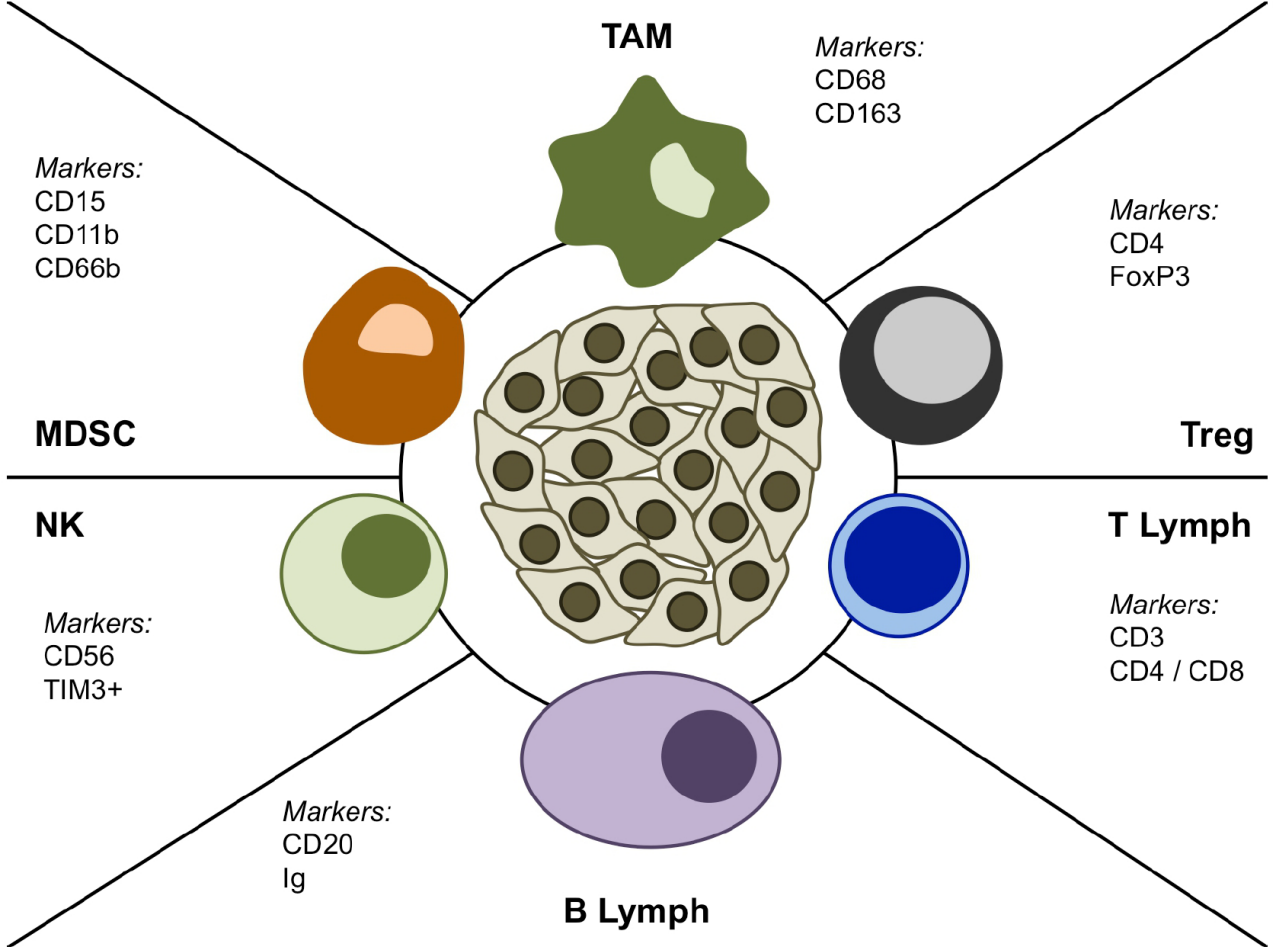
Schematic representation of the main cellular populations in the PM microenvironment.

The use of specific biomarkers such as CD68 and CD163 (**Figure 1**) indicating the presence of immunosuppressive macrophages was associated with poor prognosis in patients with epithelioid PM (23). Similarly, high levels of circulating monocytes are correlated with worse survival after cytoreductive surgery (14). Low lymphocyte-to-monocyte ratio in peripheral blood from PM patients is also a negative prognostic factor (24).

### Myeloid-derived suppressor cells

Myeloid-derived suppressor cells (MDSCs) are heterogeneous immature myeloid cells, which can expand during chronic inflammation and usually increase as a consequence of tumor progression. MDSCs can be grouped in two main subpopulations: polymorphonuclear (PMN-MDSCs) or monocytic (M-MDSCs) (25). The distinction between MDSCs and other immune cells such as TAMs has not been yet standardized. So, these cells are included in the neutrophilic infiltrate, which has a known unfavorable prognostic role and is characterized via IHC by using CD66b and CD15 (26, 27). In the PM microenvironment neutrophils can acquire a phenotype, consistent with PMN-MDSCs, which is characterized by the expression of CD15+, CD11b+, CD66b+ (**Figure 1**), and absence of CD14/CD33 (28). However, increased levels of CD11b+CD15+HLADR- neutrophils were also found in the peripheral blood from mesothelioma patients (27). Both a greater tumor-associated neutrophilic infiltrate and increased levels of peripheral blood neutrophils are associated with a worse prognosis in epithelioid MPM (29). Recently, a prognostic role in PM patients was observed for both PMN-MDSCs and M-MDSCs from pleural fluids and tissues (30).

Though targeting MDSCs could seem an interesting therapeutic approach to explore, MDSCs-directed agents such as the PI3k γ/δ inhibitor eganelisib (IPI-549) have not produced convincing clinical benefit (31).

### T lymphocytes

The immune cell infiltrate of mesothelioma includes 20–40% T-lymphocytes, mainly CD8+ T-cells, but also CD4+ and CD4+ FoxP3+ T-cells (**Figure 1**) (32, 33). The function of T lymphocytes is regulated by both neo-antigenic stimuli and checkpoint molecules. The presence of neoepitopes can be identified via next generation sequencing (34). CD8+ T lymphocytes in pleural effusions highly express CD25+ as a marker of activation (20). Moreover, perforin expression in CD8+ T-cells was related to the number of neoepitopes (35). Apart from these markers of activation, an exhausted phenotype (PD-1+, TIM3+, and LAG3+) can also be found (36). Interestingly, the number of T lymphocytes in mesothelioma tissue can influence prognosis; along this line, CD4+ T lymphocytes and CD8- T cells are associated with a better and poorer survival of PM patients, respectively (36–38).

### B lymphocytes

B lymphocytes can be found in both tumor tissue and stroma from mesothelioma patients and a higher number of B lymphocytes correlates with a better prognosis (37, 39). Additionally, these cells produce autoantibodies, e.g., those targeting the nuclear fraction, which were found in some mesothelioma patients (40). The subclasses of antibodies produced by B lymphocytes from tissue of mesothelioma patients are mainly IgG1 and IgG3, and they are already known to activate complement (**Figure 1**) (41). However, poor information on B-cell-related cytokines and B-regulatory cells is available in mesothelioma (42). More recently, lymphoid aggregates or tertiary lymphoid structures (TLS) have been identified in PM microenvironment, suggesting their potential prognostic role. Along this line, Mannarino and collegues, in a retrospective multicenter cohort of 129 chemo-naive epithelioid PM patients, demonstrated that long-term survivors (>36 months) had a specific inflammatory background with a higher number of B lymphocytes and a prevalence of TLS formations compared to short survivors (<12 months) (43). These findings further underline that PM patients, even within the same histological subtype, can have a very different clinical outcome, and demonstrate that the TME and its multiple cellular components can play a role in the clinical course of PM.

### Natural Killer cells

Small amounts of CD3-CD56+ Natural Killer (NK) and CD3+CD56+ Natural Killer T (NKT) cells were observed in mesothelioma tissues (**Figure 1**) (44, 45). In pleural effusions these cells are characterized by both the inhibitory receptor NKG2A and the activating receptor NKG2D. In peripheral blood they also express the exhaustion marker TIM3+ (**Figure 1**) (45). Evidences demonstrated that treatment with IL-2 *in vitro* can restore the cytotoxicity of NK cells from malignant effusions (46). However, NK cells do not seem the key players in the mesothelioma microenvironment, because neither NK cell depletion *in vivo* nor reduced NK cell function are able to impair tumor growth (47). Similarly, NK cell detection via immunohistochemistry is not related to change in the prognosis of mesothelioma patients (37).

## ICI-based immunotherapy

Since 1980s, evidence of clinical activity of immunotherapeutic agents, mostly interleukin-2, have been reported in highly selected mesothelioma patients; however, the clinical exploitation of these agents was limited due to their relevant toxicity or inefficacy observed in a large proportion of patients.

In the last decade, a better knowledge of the immunobiology of tumor cells and of their interactions with immune system, led to the finding that silenced immune responses can be re-activated by targeting immune checkpoint molecules expressed on T cells, with a consequence to promote efficient antitumor responses. This approach proved to be successful ushering in a new era in cancer treatment, and it is now considered the gold standard regimen in a variety of tumors, including PM. Indeed, treatment with nivolumab plus ipilimumab demonstrated a significant improvement in survival over the platinum-based regimen in the Phase III CheckMate 743 study (8), quickly leading to the approval of the immunotherapy doublet, which is now the first therapeutic choice for patients with advanced PM in most countries.

Meaningfully, the successes of immune checkpoint blockade in early lines for PM patients followed a long course of early phase studies with CTLA-4/PD-1/PD-ligand(L)-1 blocking drugs that demonstrated initial signs of antitumor activity in pretreated PM patients (48).

### Targeting CTLA-4

The phase II study MESOT-TREM 2008 represents the pioneer study that opened the path toward ICI therapy in PM patients. In this trial, 29 pre-treated PM and peritoneal mesothelioma patients received the anti-CTLA-4 mAb tremelimumab at 15 mg/kg every 90 days until disease progression or unacceptable toxicity. Despite the low ORR (6.9%), signs of antitumor activity were observed, particularly in terms of mOS (10.7 months) (48). Prompted by these results, the subsequent phase study II MESOT-TREM 2012 explored the efficacy of an intensified schedule of tremelimumab (10 mg/kg every four weeks for six cycles, followed by maintenance every 12 weeks) in pre-treated PM or peritoneal mesothelioma patients. Opposite to MESOT-TREM-2008, in the MESOT-TREM-2012 trial the primary endpoint was reached: the immune-related ORR was 13.8%, and a good safety profile was observed, as only 7% of patients experienced grade 3 or 4 immune-related side effects (49). Interestingly, in both MESOT-TREM trials, tremelimumab induced significant changes of selected T cell subpopulations in the peripheral blood. In particular, just after 1 or 2 cycles of treatment, a significant increase of circulating CD4+ICOS+T cells was observed and it correlated with a better survival.

Based on the promising results generated from the MESOT-TREM studies, 568 pre-treated pleural or peritoneal mesothelioma patients were enrolled in the phase IIb, placebo-controlled, DETERMINE study. In this trial, patients were randomized to receive tremelimumab at the same intensified dose given in the MESOT-TREM- 2012 study, or placebo. Unfortunately, tremelimumab failed to demonstrate an improvement in OS compared to placebo (7.7 and 7.3 months, respectively; HR = 0.92; *P* = 0.41) (50). However, notably a retrospective analysis, showed that the subgroup of patients with a higher exposure to tremelimumab had an improvement in survival than those who received placebo (51).

The most representative studies based on anti-CTLA-4 mAb are reported in **Table 1**.

**Table 1:** Representative studies with immune checkpoint inhibitors agents in PM.

TRIAL REGISTRATION NUMBER	TRIAL	YEAR	PHASE	COHORT	TREATMENT	SCHEDULE	ORR (%)	mOS (months)	TRAEs or TEAEs	Ref
NCT01649024	MESOT-TREM 2008	2009-2012	II	29 MPM and peritoneal malignant mesothelioma patients	anti-CTLA-4 tremelimumab	15mg/kg every 90 days for 4 doses	6,9	10,7	27 (93%) G 1-2 TEAE 4(14%) G 3-4 TEAE	(48)
NCT01655888	MESOT-TREM 2012	2012-2013	II	29 pretreated MPM or peritoneal mesothelioma patients	anti-CTLA-4 tremelimumab	10 mg/kg every four weeks for six cycles, followed by maintenance every 12 weeks	13,8		G 1-2 trAEs 26 (90%) G 3-4 trAEs 2 (7%)	(49)
NCT01843374	DETERMINE	2013-2014	IIB	568 pre-treated MPM or peritoneal mesothelioma patients	anti-CTLA-4 tremelimumab or placebo	10 mg/kg every four weeks for six cycles, followed by maintenance every 12 weeks	4,5 vs 1,1	7,7 vs 7,3	Grade ≥3 246 (65%) 380 (tremelimumab) 91 (48%) 189 placebo dyspnoea 34 (9%) tremelimumab vs 27 (14%) placebo diarrhoea 58 (15%) 1 (<1%), colitis 26 (7%) vs none).	(50)
NCT02054806	Keynote 028	2016	Ib	25 MPM patients	anti-PD1 pembrolizumab	pembrolizumab (10 mg/kg every 2 weeks)	20	18	16 (64%) trAE; fatigue 6 (24%), nausea 6 (24%), arthralgia 5 (20%). 5 (20%) G3 trAEs. 3 (12%) dose interruption 1 (4%) G 3 rhabdomyolysis + G2 hypothyroidism; 1 (4%) G3 iridocyclitis, grade; 1 (4%) erythema multiforme, G 3 erythema; G 2 infusion-related reaction.	(52)
n.a.	MERIT II	2016	II	34 pre-treated PM patients	anti-PD1 nivolumab	flat dose of 240 mg biweekly	29	17,3	32 (94%) AEs 26 (76%) TRAEs.	(53)
NCT02991482	PROMISE-Meso	2017-2018	III	144 pre-treated PM patients	anti-PD1 pembrolizumab or chemotherapy	pembrolizumab (200 mg/Q3W) vs single-agent chemotherapy (gemcitabine or vinorelbine)	22 vs 6	10,7 vs 12,4 not significant	G ≥3 TRAE 19.4% (pembrolizumab), 1 G3 hypophysitis, 25.7% (chemotherapy)arm5 treatment discontinuations per arm	(54)
NCT02588131	NIBIT-MESO-1	2015-2016	II	40 MPM patients	anti-CTLA-4 tremelimumab + anti-PD-L1 durvalumab	tremelimumab, given at 1mg/kg + durvalumab given at 20 mg/kg, every four weeks	28	16,6	75% trAEs 18% had G3-4 trAEs	(48, 55)
NCT02716272	MAPS-2	2016	II	125 pre-treated MPM patients	anti-CTLA-4 ipilimumab + anti-PD-1 nivolumab or nivolumab	nivolumab (3 mg/kg biweekly) vs nivolumab (3 mg/kg biweekly) ipilimumab (1 mg/kg every 6 weeks)	29	11,9 (nivolumab) 15,9 (nivolumab + ipilimumab)	9/63 nivolumab G3/4 16/61 nivolumab + ipilimumab	(56)
NCT02899299	CheckMate 743	2016-2018	III	605 first-line MPM patents	anti-PD-1 nivolumab + ipilimumab vs platinum plus pemetrexed chemotherapy	nivolumab (3 mg/kg biweekly) ipilimumab (1 mg/kg every 6 weeks)	40 vs 43	18,1 vs 14,1. (16,5 vs 8,8 in non-epithelioid histology).	G3-4 trAEs 30% nivolumab plus ipilimumab 32% of 284 chemotherapy. 3 (1%) treatment-related deaths occurred nivolumab plus ipilimumab (pneumonitis, encephalitis, and heart failure) 1 (<1%) chemotherapy group (myelosuppression).	(8)
ACTRN12616001170415	DREAM	2016-2017	II	54 MPM patients	Cisplatin + pemetrexed + anti-PD-L1 durvalumab	cisplatin (75 mg/mq) + pemetrexed (500 mg/mq) + durvalumab (1125 mg) Q3W for six courses, then maintenance with durvalumab	48	18,4	Most commonG3-4 AEs: neutropenia (13), nausea (6 11%) anaemia (4/7%).	(57)
NCT02899195	PrE0505	2017-2018	II	55 MPM patients	anti-PD-L1 antibody durvalumab plus platinum-pemetrexed chemotherapy	Durvalumab (at a fixed dose of 1,120 mg intraveneously) given once every 3 weeks in combination with pemetrexed and cisplatin at their standard doses for up to six cycles	56,4	20,4	G2 or lower irAEs	(58)
UMIN000030892	JME-001	2018-2019	II	18 MPM patients	anti-PD-1 nivolumab and cisplatin + pemetrexed.	Cisplatin (75 mg/m2), pemetrexed (500 mg/m2), and nivolumab (360 mg/body) intravenously every 3 weeks (4-6 cycles).	77,8	20,8	55.6% G3	(59)
NCT02784171	IND.227	2017-2020	III	440 MPM patients	anti-PD1 pembrolizumab +platinum based chemotherapy vs platinum based chemotherapy	222 (pembrolizumab + chemotherapy) 218 platinum based chemotherapy	62% in pembrolizumab + chemotherapy arm 38% in chemotherapy arm	17,3 months (95% CI 14,4-21,3) (pembrolizumab + chemotherapy) Vs 16,1 months (95% CI 13,1-18,2) in chemotherapy arm	28% in pembrolizumab + chemotherapy arm 16% in chemotherapy arm	(60)
NCT04334759	DREAM3R	2020-2025	III	enrolling 480 MPM patients	durvalumab + chemotherapy vs chemotherapy vs ipilimumab plus nivolumab.	Durvalumab 1500 mg Q3W+ Cisplatin 75 mg/m² or Carboplatin AUC 5 Q3W + Pemetrexed 500 mg/m² IV Q3W (4 -6 cycles), maintenance Durvalumab 1500 mg IV Q4W	Results expected in late 2025			

### Targeting PD1/PDL1 axis

On the wave of the success obtained with the PD-1/PD-L1 blocking mAb in metastatic melanoma patients, and then in other tumor types, great interest has been addressed to investigate their efficacy also in PM patients, due to their more favorable efficacy and safety profile compared the anti-CTLA-4 mAb. Consistently, a variety of phase I/II trials were conducted in PM patients (52, 53, 61–65). Among the most representative studies, the phase Ib Keynote 028 study (NCT02054806) explored the efficacy of pembrolizumab in 25 PM patients showing a response rate of 20% and a mOS of 18 months (52). Promising results were also observed in the MERIT II study, which led to the approval of nivolumab in second-line PM patients in Japan. Indeed, in the study, 34 pre-treated PM patients received nivolumab at the flat dose of 240 mg every two weeks; results showed a mOS of 17.3 months, three-year survival of 23.5%, mPFS of 6.1 months, and ORR of 29%, regardless of the mesothelioma histotype (61).

Following these exciting results, two phase III studies, the PROMISE-Meso and the CONFIRM trials were started (54, 66). In the first one, 144 pre-treated PM patients were randomized to receive chemotherapy (gemcitabine or vinorelbine) or pembrolizumab. Crossover to pembrolizumab was allowed. Unfortunately, no improvement in mOS (10.7 *vs*. 12.4 months; HR = 1.12; 95% CI: 0.74-1.69; *P* = 0.59) and mPFS (2.5 *vs*. 3.4 months; HR = 1.06; 95% CI: 0.73-1.53; *P* = 0.76) was observed with pembrolizumab over chemotherapy. However, in patients treated with pembrolizumab, an increase in response rate was reported (22% *vs*. 6% treated with chemotherapy, *P* = 0.004) (54). In the CONFIRM study, 332 second- or third-line PM patients were randomized to receive nivolumab or placebo. In the trial, cross-over was not permitted. Median OS was higher with nivolumab than in the placebo group (9.2 *vs*. 6.6 months; HR = 0.72; 95% CI: 0.55-0.94; *P* = 0.018). The improvement in OS was seen in patients with epithelioid histology (9.4 *vs*. 6.6; HR = 0.71; 95% CI: 0.53-0.95; *P* = 0.021) but not in those with non-epithelioid histology (5.9 *vs*. 6.7 months; HR = 0.79; 95%CI: 0.35-1.79; *P* = 0.572). The grade 3 and 4 treatment-related adverse event rates were 13.1% in the nivolumab arm and 2.7% in the placebo arm (66).

Among predictive biomarkers of response to PD1/PD-L1 blockade studied, the tumor expression of PD-L1 was largely investigated in the studies above reported, with unconclusive results (67).

### Co-targeting CTLA-4 and PD-L1/PD-L1 axis

Though single agent ICI demonstrated a meaningful clinical benefit in PM in several early phase trials, the major limitation of these studies was represented by both the small number of enrolled patients and the low number of objective responses achieved. Thus, the investigation of the role of ICI in PM reached a turning point by exploring the co-targeting of CTLA-4 and of the PD1/PD-L1 axis, increasing the efficacy of ICI therapy and overcoming the mechanisms of resistance in tumor cells. This change of direction of clinical research has proved successful.

Along this line, the NIBIT-MESO-1 was the first study investigating the efficacy of an anti-CTLA-4 combined with an anti-PD L1 mAb. In this pivotal single arm phase II trial, tremelimumab, given at 1mg/kg, was combined with the anti-PD-L1 durvalumab, at 20 mg/kg, every four weeks for the first four courses of treatment (induction phase) and then as a single agent for additional nine courses at the same dose (maintenance phase). The study protocol foresaw the possibility of retreatment with durvalumab and tremelimumab in patients who had initially achieved a clinical benefit and that subsequently progressed. The primary endpoint of the trial was ORR assessed *per* immune-related (ir) RECIST, while secondary objectives were ORR, PFS *per* modified RECIST, OS, and safety. At a median follow up of 19.2 months, irORR was 28%, irPFS was 8 months, while PFS was 5.7 months and OS 16.6 months (48). An updated survival analysis reported 20% and 15% of the patients alive at 3- and 4 years, respectively (55). Interestingly, the NIBIT-MESO-1 was also the first study to prospectively explore the efficacy of combo-ICI retreatment. Consistently, 17/40 patients were eligible for re-treatment as *per* protocol. Though no ir-ORR was achieved, a disease control was achieved by the 40% of patients; in addition, a noteworthy 1-year survival from the starting of retreatment was observed in 52.9% of subjects (55). Notably, patients who benefitted from immunotherapy retreatment showed a TMB above the median than patients who did not achieve a clinical benefit from retreatment (55). Though this finding requires caution being generated in a small proportion of patients, it underlines the potential predictive role of the TMB for ICI rechallenge (55). The promising results generated in the NIBIT-MESO-1 study, were then confirmed in two additional phase II studies, the INITATE and the MAPS-2, investigating the combination of ipilimumab and nivolumab in pre-treated PM patients. The first trial, INITIATE (68), was a phase II trial in which patients with recurrent mesothelioma were treated with nivolumab (240 mg every 2 weeks) plus ipilimumab (1 mg/kg every 6 weeks). The primary endpoint of the trial was Disease Control Rate (DCR) at 12 weeks, while OS, PFS, and RR were secondary endpoints. Thirty-six patients were treated with nivolumab plus ipilimumab in this trial, DCR at 12 weeks was 68%, ORR was 29%, median PFS was 6.2 months while mOS was not reached at the time the results were published, but the estimated mOS was more than 12.7 months. In the phase II, non-comparative MAPS-2 study (56), patients with refractory mesothelioma were randomized to receive either nivolumab (3 mg/kg q2w) or nivolumab plus ipilimumab (1 mg/kg q6w). Primary endpoint of the trial was DCR at 12 weeks, while PFS, OS and ORR were secondary endpoints. Sixty-two patients were treated in the nivolumab plus ipilimumab arm, 12-weeks DCR was 50% of ipilimumab plus nivolumab, ORR was 29% while mPFS was 5.7 months and mOS was 15.9 months. Due to the non-comparative design of the trial, no formal comparison between the nivolumab and the nivolumab plus ipilimumab arms was possible, however, DCR, PFS, ORR and OS were all numerically higher in the nivolumab plus ipilimumab arm than in that with nivolumab alone.

The results generated from the NIBIT-MESO-1, MAPS-2, and INITIATE studies strongly contributed to the activation of the phase III, multicenter, randomized, CheckMate 743 trial (8). In the study, 605 first-line PM patients were randomized with a 1:1 ratio to receive either nivolumab plus ipilimumab or platinum-based chemotherapy; the primary endpoint of the trial was OS in the overall population, key secondary endpoints were PFS, ORR, and safety. At a median follow up of 29.7 months, OS in the ipilimumab plus nivolumab arm was significatively longer than in chemotherapy arm (18.1 vs 14.1 months, HR 0.74, 96,6% CI 0,6–0,9; p=0,002). Conversely, mPFS (6.8 vs 7.2 months, HR 1, 95% CI 0.82-1.2) and ORR (40% vs 43%) were statistically similar between the two study arms, but notably five complete responses occurred in the ipilimumab plus nivolumab arm. Considerably, the most relevant survival benefit with the dual IC was observed in the non-epithelioid histotype, compared to that observed with chemotherapy (16.5 vs 8.8 months, HR 0.46 95% CI 0.3-0.7). This result has led to the approval of the dual ICI regimen in several countries but only in the non-epithelioid histology in Italy. Interestingly, the 3 and 4-years update largely confirms the survival benefit with nivolumab plus ipilimumab depicted in the primary analysis (23% and 17% respectively at 3- and 4 years) compared with chemotherapy (15% and 11%, respectively at 3- and 4 years). At an exploratory analysis investigating the expression of CD8A, STAT1, LAG3, and CD274 (PD-L1) by using RNA sequencing, a high four-gene inflammatory signature score associated with an OS improvement in the dual ICI arm (mOS 21.8 months versus 16.8 months in patients with low score). Conversely, no correlation between inflammatory gene signature score and response was identified in the chemotherapy arm (69). Therefore, inflammatory signature score seems to represent a potential predictive biomarker of response to ICI combination.

The logical next step was the exploration of ICI plus chemotherapy. Indeed, preclinical evidences in murine models of mesothelioma showed that chemotherapy in combination with immunotherapy increased the tumoral CD4+/CD8+ immune infiltrate and induced long term tumoral responses (70, 71). Along this line, a variety of phase II/III trials have explored the efficacy of platinum-pemetrexed regimen in combination with ICI in first-line PM patients. In the single arm, phase two, DREAM trial (57), 54 patients received the combination of cisplatin (75 mg/mq) plus pemetrexed (500 mg/mq) combined with durvalumab (1125 mg) every three weeks for six courses, then durvalumab alone for 12 months (maintenance phase). The primary endpoint of the trial was 6 months PFS, while ORR, PFS and OS were secondary endpoints. Thirty-one out of 54 patients (57%) were 6-months progression-free, 48% subjects had an ORR, mPFS was 6.9 months according to mRECIST, and mOS 18.4 months. In the phase II PrE0505 study (58), 55 patients were enrolled and received carboplatin or cisplatin plus pemetrexed and durvalumab (1100 mg) every three weeks and subsequently durvalumab maintenance up to 12 months. At a median follow up of 24.2 months, the mOS (primary endpoint of the study) of 20.4 months was significatively longer than historical controls (12.4 months, HR 0.34 p 0.0014). Among secondary endpoints, mPFS was 6.7 months and ORR 56.4%.

In the JME-001 trial (59), 18 patients received in the first line the combination of nivolumab (360 mg q3w) and cisplatin plus pemetrexed. The trial primary endpoint was ORR assessed per mRECIST, while mPFS, OS and duration of response (DOR) were secondary endpoints. Fifteen patients had an objective response (77.8%) with a mDOR of 6.7 months, the median PFS was 8.0 months, and mOS 20.8 months. Interesting results were also reported at the interim analysis of the phase II IND-227 trial (72), that investigated the efficacy of pembrolizumab alone or in combination with platinum-based chemotherapy compared to chemotherapy alone. In the study, patients treated with pembrolizumab plus chemotherapy achieved a mPFS of 6.8 months, a mOS of 19.2 months, and an ORR of 48%. The promising results observed in the phase II, lead to the activation of the phase III study investigating the combination of pembrolizumab in combination with chemotherapy versus standard first line chemotherapy. The updated results of the phase III part of the trial demonstrated a significant improvement in OS (17.3 vs 16.1 months, respectively, *p <*0.03, HR=0.79, p =0·0324) in the combination group (60). In addition, in the combination arm 62% of the patients achieved an objective response and only 2% of the patients were primary progressors (60).

Finally, two phase III studies, the DREAM3R trial (NCT04334759) and the BEAT-Meso trial (NCT03762018) are investigating the efficacy of chemotherapy alone versus durvalumab with chemotherapy, or atezolizumab plus bevacizumab combined with chemotherapy, respectively. Both studies are currently recruiting.

The most representative studies based on anti-PD-1/PD-L1 mAb are reported in **Table 1**.

## Novel therapeutic approaches to enhance the efficacy of ICI-based immunotherapy: the role of epigenetics in PM

Despite the accumulation of novel insights about mesothelioma biology and early excitement regarding the promise of ICI, the majority of PM patients fails to derive clinical benefit from or ultimately develop resistance to such treatment (73). Thus, the development of novel combinatorial strategies with ICI is needed to maximize clinical benefit. In this context, priming the immune system with epigenetic therapy is an emerging paradigm and an area of active clinical investigation also in PM. Specifically, epigenomic signatures in immune and cancer cells appear to be a promising predictor of clinical outcome for immunotherapy treated patients. Besides, considering the established role played by epigenetics in PM initiation and progression, the investigation of the epigenetic-based immunotherapies seems to have a relevant potential to increase the management of PM patients (74, 75).

Several studies have investigated the DNA methylation profile in mesothelioma cells in order to better understand the role of epigenetics in this malignancy. For example, Christensen and colleagues (76) found that, compared to non-tumor pleura, PM cells had a distinct methylation profile, which could be used to distinguish mesothelioma from normal cells. This study also found that the DNA methylation profile was significantly associated with lung asbestos burden and clinical outcome, suggesting that epigenetic alterations may be important in mesothelioma development and progression (76). Furthermore, the methylation level of CpG sites is associated also with PM histology: CpG sites whose methylation level correlated with the sarcomatoid PM were preferentially located in CpG islands, in contrast to those whose methylation levels correlated with the epithelioid PM, which were mainly located in non-CPG islands (77).

Epigenetic changes have been also shown to play a key role in the resistance to immunotherapy in PM (78). One way in which epigenetic changes can contribute to resistance is by downregulating the expression of genes involved in antigen processing and presentation which impaired the ability of the immune system to recognize and attack the cancer cells. Another way in which epigenetic changes can contribute to resistance is by upregulating the expression of immune checkpoint proteins, such as PD-L1, which can inhibit the activity of T cells, allowing cancer to evade the immune response. Epigenetic changes can also affect the function of immune cells themselves, impairing the differentiation and function of T cells trough the reduction of genes expression involved in CD8+ T cell differentiation and function, such as the transcription factor T-bet, cytokines interferon gamma (IFN-γ), and tumor necrosis factor alpha (TNF-α). These modifications render T cells less effective at recognizing and attacking cancer cells (79).

To reverse epigenetic changes that impair the immune response and overcome resistance to immunotherapy in mesothelioma, researchers have investigated the use of epigenetic drugs, such as DNA hypomethylating agents (DHA) and histone deacetylase inhibitors (HDACi), providing insights into the underlying mechanisms of how epigenetic drugs can enhance the effectiveness of immunotherapy. Among different available epigenetic drugs, DHA represents a promising enhancer of immunogenicity of PM cells and a potential inducer of increased immune cell recognition of tumor cells. Several preclinical studies demonstrated that epigenetic remodelling of cancer cells by DHA, in particular decitabine and guadecitabine, induced/up-regulated the expression of different immune-related molecules (i.e., HLA class I, cancer testis antigens (CTA), co-stimulatory molecules, interferon stimulated genes) in cancer cells of different histotypes including PM (80–82) resulting in their improved recognition by immune cells (83–87). DHAs were also demonstrated to sensitize PM cells to the modulation of immune response through the upregulation of several genes involved in crosstalk between dendritic cells and NK cells signaling, dendritic cell maturation and acute phase response signalling (87). In addition to DHA, also HDACi, valproic acid (VPA) and vorinostat (SAHA), were investigated and demonstrated to synergized with decitabine to kill PM cells and induce tumor antigen expression in the remaining living tumor cells. As a consequence, tumor cells expressing these antigens were recognized and lysed by specific CD8+ cytotoxic T-cells. Moreover, *in vivo* treatment with decitabine and VPA inhibited tumor growth, and promoted lymphocyte infiltration and an immune response against PM cells (88). More recently, new HDACi in pre-clinical development have shown that they can induce PD-L1 in PM cell lines *in vitro* (89), suggesting a HDACi as potential partner in a combinatorial immunotherapeutic approach in mesothelioma. These observations led to the development of a Phase I study in which PM patients were treated with the HDACi, SAHA. The results of this study showed a partial response in 2 out of 13 patients (90); however, no improvement in OS was demonstrated in a subsequent phase III trial in which 650 PM patients were treated with SAHA (91).

Immunomodulatory activities of epigenetic drugs are not limited to DHA or HDACi; indeed, also enhancer of zeste homolog 2 inhibitors (EZH2i) have been demonstrated to have a key role in the PM-immune system crosstalk. As recently shown by Hamaidia et al., the inhibition of EZH2 reduced cytotoxic effects of macrophages toward PM cell lines through the up-regulation of PD-1 on macrophage surface; thus, the concomitant inhibition of EZH2 and PD-1 could restore immunoediting activity of macrophages (92). These data could justify the design of clinical trials combining anti-PD-1 mAbs and EZH2i, and in general ICI and epi-drugs, to explore PM innovative epigenetic-based immunotherapy for this still hard-to-treat tumor.

These studies suggest that epigenetic inhibitors may increase the efficacy of immunotherapy by enhancing antigenicity and presentation of tumor-associated antigens, reprogramming the tumor microenvironment to counteract immunosuppressive mechanisms, and reversing cytotoxic T cell exhaustion. The therapeutic potential of combining epigenetic therapies with immunotherapy was first indicated by reports demonstrating that immune or inflammatory-related gene signatures were increased upon inhibition of epigenetic mechanisms (93, 94). First-in-human evidence of this previously unexplored strategy has been provided by the phase Ib NIBIT-M4 trial (NCT02608437), in which patients with unresectable melanoma were treated in a sequential schedule with the DHA guadecitabine followed by ipilimumab. The combination demonstrated to be safe and tolerable, and analysis of the tumor-immune contexture demonstrated the up-regulation of immune-related molecules, such as HLA class I, and an increase in CD8+T cells infiltration (95), as well as re-expression of immuno-modulatory endogenous retroviruses and other repetitive elements (96).

In this context, in mesothelioma mouse models, preclinical studies investigating the use of decitabine in combination with the ICI anti-CTLA-4 demonstrated that the combined therapy improved the anti-tumor activity compared to each treatment alone, and also led to increased infiltration of T cells into the tumors (85).

In summary, epigenetic drugs have the potential to enhance the effectiveness of immunotherapy in PM by reversing epigenetic changes that impair the immune response and by improving the function of immune cells. These drugs may offer a promising strategy for improving the outcomes of mesothelioma patients receiving immunotherapy.

The most representative studies based on combination ICI regimens are reported in **Table 1**.

## Non-ICI based immunotherapy

### Unlocking the power of dendritic cell vaccination for improving survival of PM patients

Dendritic cells (DCs) are the most potent and specialized antigen presenting cells (APCs) that play a crucial role in initiating and regulating a primary T-cell immune response. DCs vaccination is a type of immunotherapy that involves harvesting DCs or their precursors from the patient’s blood or bone marrow to be differentiated *ex vivo*, expose them to cancer cells or cancer antigens *in vitro*, and then injecting them back into the patient to stimulate an immune response against tumor cells. This process primes the DCs to recognize and present cancer antigens to the immune system and activates antigen- specific T cells that can migrate into the tumor, recognize and attack cancer cells.

Since 2004, *in vitro* data have demonstrated that DCs pulsed with apoptotic PM cells can elicit cytotoxic T-lymphocyte (CTL) responses, thus raising the idea that DC immunization could be an effective treatment for PM (97). Further investigations confirmed that this strategy could inhibit tumor growth and boost antitumor immunity in mice models of PM (98).

**Figure 2:**
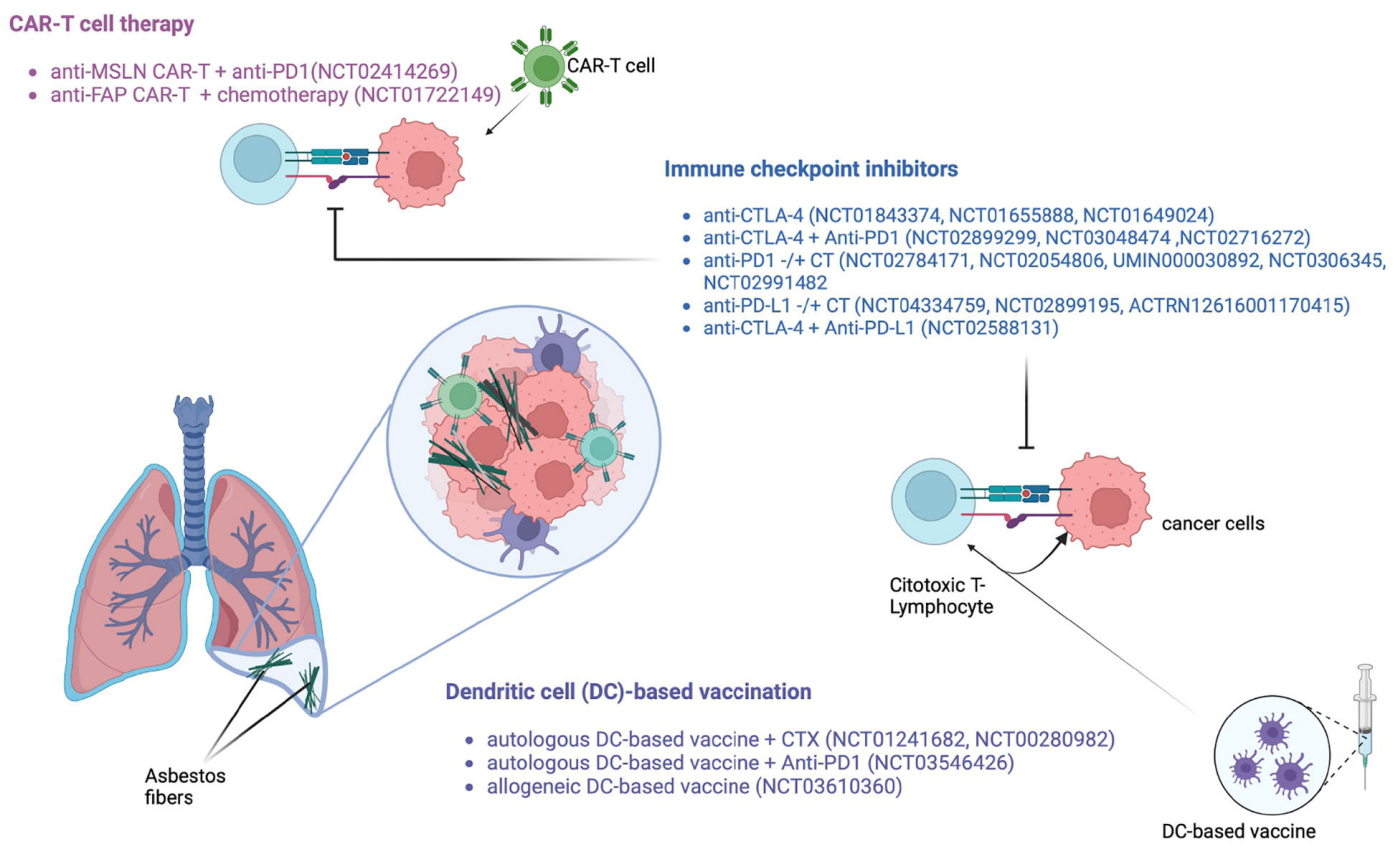
Main immunological approaches under investigation for PM patients.

Several clinical trials have later reported promising results in improvement in survival with DC vaccination in PM patients. Consistently, in a phase I trial, recruiting 10 PM patients (NCT00280982) (**Figure 2**), treatment with DC vaccination was well-tolerated and resulted in a mOS of about 19 months (78). Further early phase I/II studies in 29 PM patients demonstrated the effectiveness of DC immunization with a mOS of 27 months, and the 2- and 5- year survival of 55.2% and 20.7%, respectively (99). Based on these promising results, the DENdritic cell Immunotherapy for Mesothelioma (DENIM; NCT03610360) study was designed (**Figure 2**) (100). The DENIM study, an open-label randomized phase II/III clinical trial which compared DC vaccination with active symptom control in patients with disease progression on first-line pemetrexed-platinum. Importantly, PM patients, enrolled in the study, were treated with DCs loaded with Allogeneic Tumor Cell Lysate (PheraLys) instead of autologous tumor lysate; results of this trial are eagerly waited.

In the PMR-MM-002 clinical trial (NCT01241682) (**Figure 2**), treatment with tumor lysate-pulsed DC as therapeutic adjuvants showed a good safety profile (101). In another study, investigating efficacy of DC immunization plus cyclophosphamide, 70% of PM patients were alive at 2 years. Interestingly, after 7 days of therapy with low-dose cyclophosphamide, a significant decrease in the percentage of Tregs in the peripheral blood of patients was observed (9.43% vs 5.51%, *p* = 0.02).

An intriguing strategy under investigation involves a combination regimen with DC vaccination and ICIs; along this line, the currently recruiting monocentric MESOVAX trial (NCT03546426) (**Figure 2**) is exploring the efficacy of autologous DC vaccination plus pembrolizumab in second line PM patients.

Overall, studies with DC immunization showed promising results in term of efficacy and safety profile in PM patients. Mild side effects of the DC vaccine include fever, mild asthenia, and flu-like symptoms, while severe immune-related adverse effects have been rarely reported.

The most representative studies based on adoptive T cell strategies are reported in **Table 2**.

**Table 2:** Representative dendritic cell or CAR- T cell based trials in PM.

TRIAL REGISTRATION NUMBER	TRIAL	YEAR	PHASE	COHORT	TREATMENT	SCHEDULE	ORR (%)	mOS (months)	TRAEs or TEAEs	Ref
NCT00280982		2010	I	10 MPM patients	Tumor lysate-loaded autologous dendritic cells	Three vaccinations of clinical-grade autologous dendritic cells intradermally and intravenously at 2-week intervals after chemotherapy. Each vaccine was composed of 50 x 10(6) mature dendritic cells pulsed with autologous tumor lysate and keyhole limpet hemocyanin (KLH) as surrogate marker.	Not Reported	19	no G3 -4	(78)
NCT01241682	PMR-MM-002	2009-2011	I	10 MPM patients	low-dose cyclophosphamide (CTX) and dendritic cell-based immunotherapy.	Patients received at least three immunizations with mature DC loaded with autologous tumor lysate and keyhole limpet hemocyanin (KLH) with a 2-week interval.Six and 12 months after the third DC vaccination, a revaccination was given to boost the immune system if enough DCs were available (fourth/fifth vaccination).Each immunization, consisting of 50 × 106 DCs, was administered intradermally and intravenously. Two 50 mg tablets/day of CTX starting 1 week before vaccination to the day of every vaccination		70%	The only side effect being moderate fever	(101)
NCT03546426	MESOVAX		I	19 MPM patients	anti-PD1 pembrolizumab + autologous DC vaccination	Autologous dendritic cells (DC) loaded with autologous tumor homogenate, 10 x7 cells ID every 3 weeks for up to six doses. Pembrolizumab 200 mg IV q3w.Interleukin-2 3 MU s.c. from day +2 to day +6 after each DC administration	Not Reported	Not Reported	Mild side effects of the DC vaccine include fever, exhaustion, and flu-like symptoms	(102)
NCT02414269	n.a.	2015-2019	I	25 MPM patients	Mesothelin-targeted chimeric antigen receptor (CAR) T-cell therapy + anti-PD1 pembrolizumab	Single dose cyclophosphamide (1,500 mg/m2). Intrapleural administration of 0.3M to 60M CAR T cells/kg.Pembrolizumab 200 mg flat dose infusion intravenously.	Not Reported	23,9 (CAR-T + pembrolizumab)	Not Reported	(102, 103)
NCT01722149	FAPME		I	4 MPM patients	fibroblast activation protein (FAP) targeting CAR T cells (CART-FAP) + chemotherapy	Adoptive Transfer of 10e6 re-directed T cells in the pleural effusion	Not Reported	Not Reported	2 thromboembolic events	(104)

## Revolutionizing mesothelioma treatment: how CAR T cells improve survival

Chimeric antigen receptor (CAR) T cell treatment is a kind of immunotherapy that alters a patient’s own immune cells so that they can detect and kill cancer cells. Firstly, the process involves leukapheresis procedure to isolate T cells from the patient’s blood. Afterwards, CARs are transferred on the surface of these cells through genetic engineering. The T cells can then recognize and attach to specific proteins on the surface of cancer cells thanks to these receptors. After being created, CAR T cells are injected back into the patient’s bloodstream. The CAR T cells search for and attack cancer cells that express the target protein once they have entered the body. Due to the target-specificity of CAR T cells, they may be able to destroy cancer cells while sparing healthy cells if the targeted antigen is highly specific for cancer cells.

Research on CAR T cell therapy for mesothelioma is still in the early stages and only few antigens like mesothelin (MSLN) (102, 103, 105), placental-like 2 alkaline phosphate (ALPPL2) (106), fibroblast activation protein (FAP) (107, 108) and MET, have been targeted and tested so far. Nevertheless, preliminary data generated from preclinical and clinical studies have so far shown promising results in term of safety and efficacy profile.

When administered intrapleurally in mice models rather than systemically, MSLN-targeted CAR T cells showed enhanced antitumor activity, and a long-term antitumor effect linked to CD4+ T cell activation was observed (102). ALPPL2 (107) and MET-targeted CAR T cells (109), also have proven effective in preclinical models of PM. Moreover, peritumoral components such as FAP, a transmembrane serine protease that is highly expressed in cancer-associated stromal cells, can be targeted by CAR T cells (104). In a phase I study (NCT02414269) investigating the efficacy of anti-MSLN CAR T cell therapy combined with pembrolizumab in PM patients revealed an ORR of 63%, a mOS of 23.9 months, and 83% 1-year OS (102, 103) (**Table 2**).

Several proteins may serve as therapeutic targets for CAR T cell therapy in PM patients, including FAP and anti-FAP CAR T cells (**Figure 2**) that have demonstrated a good safety profile and to be able to expand in the peripheral blood of patients after intrapleural administration (107). CAR T cell therapy can cause side effects, including cytokine release syndrome (CRS) and neurotoxicity. CRS is a systemic inflammatory response that can cause fever, chills, low blood pressure, and organ dysfunction. Immune effector cell-associated neurotoxicity syndrome (iCANS) can cause confusion, seizures, and other neurological symptoms. However, these side effects that are typically reported in hematological malignancies, are manageable and can be treated with medications. Side effects with CAR T cells in PM patients have been so far rare and properly tackled.

Overall, though CAR T cell treatment has shown in early phase studies to prolong OS and PFS of PM patients, the exploitation of CAR T cell strategy is still in its infancy in this disease. Further studies in large cohorts of PM patients will be needed, as well as a deep comprehension of their interactions with tumor microenvironment components to boost CAR T potency, also by utilizing them within appropriate combination regimen.

## Conclusion and future directions

For decades, no relevant progress has been made in PM treatment, and many drugs investigated alone or in combination regimen have failed to demonstrate efficacy. A better knowledge of tumor immunology and the role of TME has recently led to a therapeutic paradigm shift also in this disease with the approval of the first chemotherapy-free regimen based on the dual ICI nivolumab plus ipilimumab.

Certainly, much has to be gained to overcome the immune resistance observed in a still large PM population. Along this line, new immunotherapeutic strategies are currently under active investigation.

A greater understanding of the complex immunological responses against the tumor, together with the identification of predictors of response to immunotherapy, continue to be expanding areas of basic and clinical research and will hopefully help to drive patient selection to such treatments. Along this line, multi-omics and AI-based approaches are becoming a key contributor to anticancer drug development, revealing new concepts for laboratory research and clinical investigation.

## Author contributions

LC: Conceptualization, Funding acquisition, Writing – original draft, Writing – review & editing. GB: Writing – original draft, Writing – review & editing. FG: Writing – original draft, Writing – review & editing. LC: Writing – original draft, Writing – review & editing. AD: Writing – original draft, Writing – review & editing. FN: Writing – original draft, Writing – review & editing. MM: Writing – original draft, Writing – review & editing. AD: Writing – original draft, Writing – review & editing. AC: Writing – original draft, Writing – review & editing. ML: Writing – original draft, Writing – review & editing. LC: Writing – original draft, Writing – review & editing. MM: Writing – original draft, Writing – review & editing, Supervision.
